# At What Price? A Cost-Effectiveness Analysis Comparing Trial of Labour after Previous Caesarean versus Elective Repeat Caesarean Delivery

**DOI:** 10.1371/journal.pone.0058577

**Published:** 2013-03-06

**Authors:** Christopher G. Fawsitt, Jane Bourke, Richard A. Greene, Claire M. Everard, Aileen Murphy, Jennifer E. Lutomski

**Affiliations:** 1 National Perinatal Epidemiology Centre, Cork, Ireland; 2 School of Economics, University College Cork, Cork, Ireland; 3 Cork University Maternity Hospital, Cork, Ireland; Consejo Superior de Investigaciones Cientifics, Spain

## Abstract

**Background:**

Elective repeat caesarean delivery (ERCD) rates have been increasing worldwide, thus prompting obstetric discourse on the risks and benefits for the mother and infant. Yet, these increasing rates also have major economic implications for the health care system. Given the dearth of information on the cost-effectiveness related to mode of delivery, the aim of this paper was to perform an economic evaluation on the costs and short-term maternal health consequences associated with a trial of labour after one previous caesarean delivery compared with ERCD for low risk women in Ireland.

**Methods:**

Using a decision analytic model, a cost-effectiveness analysis (CEA) was performed where the measure of health gain was quality-adjusted life years (QALYs) over a six-week time horizon. A review of international literature was conducted to derive representative estimates of adverse maternal health outcomes following a trial of labour after caesarean (TOLAC) and ERCD. Delivery/procedure costs derived from primary data collection and combined both “bottom-up” and “top-down” costing estimations.

**Results:**

Maternal morbidities emerged in twice as many cases in the TOLAC group than the ERCD group. However, a TOLAC was found to be the most-effective method of delivery because it was substantially less expensive than ERCD (€1,835.06 versus €4,039.87 per women, respectively), and QALYs were modestly higher (0.84 versus 0.70). Our findings were supported by probabilistic sensitivity analysis.

**Conclusions:**

Clinicians need to be well informed of the benefits and risks of TOLAC among low risk women. Ideally, clinician-patient discourse would address differences in length of hospital stay and postpartum recovery time. While it is premature advocate a policy of TOLAC across maternity units, the results of the study prompt further analysis and repeat iterations, encouraging future studies to synthesis previous research and new and relevant evidence under a single comprehensive decision model.

## Introduction

Whether women should attempt a trial of labour after caesarean (TOLAC), rather than undergo an elective repeat caesarean delivery (ERCD) is an important clinical decision. While TOLAC is a viable birth option for many low risk women and is associated with numerous benefits relative to ERCD [1], [2], attempted TOLAC rates vary dramatically across local hospitals and internationally [3]–[7]. ERCD may be favoured over a trial of labour due to medico-legal fears [8], [9], potential risks to the mother and fetus [10]–[12], or maternal preference [13]. However, the increased frequency of elective caesarean delivery carries many important economic implications. The cost to the health system is typically greater than the cost of vaginal deliveries, and the impact on a woman’s health related quality of life following the surgical intervention is considerably more profound [2], [14].

Costing mode of delivery is challenging. Hospital charges are often cited as indication of the cost of care; however, these standard charges rarely reflect the actual cost of providing a service [15]. The actual cost of providing a service is best estimated by identifying, measuring, and valuing all resources used in the production of the service [16], known as the “bottom-up” approach or micro-costing. Internationally, attempts have been made to estimate the delivery costs associated with a vaginal and caesarean delivery by determining direct medical costs [17]; direct and indirect medical costs, and fixed and variable costs [18]; hospital charges [19], [20]; cost-to-charge ratios [21]; and per diem rates [22], with some studies including physician fees in their analysis [21], [22]. However, due to this wide variety of costing techniques, these studies have reached divergent conclusions regarding estimated mode of delivery costs. Whereas estimates in the UK have found that a caesarean delivery costs more than double a vaginal delivery [23], two studies conducted in the US have contradicted this finding [17], [20]. For instance, Kazandjian *et al* found that the average cost of a vaginal delivery in the US may be higher than the average cost of a caesarean delivery when certain maternal characteristics are accounted for, such as maternal race and the presence of maternal co-morbidities [21]. This study contributes to and enhances existing costing estimations. It applies a “bottom-up” approach in its evaluation of both delivery procedures, accounting for medical supplies, pharmaceuticals, and staff costs. The exhaustive costing technique provides invaluable information on the total cost of care following a TOLAC and ERCD expressed in Euros.

The obstetric course of women with a previous uterine scar attracts major public policy concern as the clinical decision does not just affect the woman and infant but also the health care system and, moreover, society as a whole. Over the last 30 to 40 years, researchers have attempted to discern the appropriate mode of delivery for women who are considered at low risk of obstetric complications. However, most studies have found conflicting evidence, with some studies suggesting that a TOLAC is associated with greater maternal morbidities than an ERCD [24]–[26] while other studies have demonstrated the opposite [2]. Few studies have incorporated the economic implications of the clinical decision in their analysis but with little appeal to public and clinical conviction [21], [22]. To determine the most suitable mode of delivery for low risk women from a public policy perspective, research must converge on a point where the level of uncertainty surrounding the expected clinical outcome is minimised [27]. This involves numerous evaluations, along with the amalgamation of previous research, or synthesis of evidence. In health economics, this approach is referred to as the iterative framework of economic evaluation [28]–[30]. Because this study represents the first of its kind in a European setting, it begins the iterative approach to determining the appropriate mode of delivery for women with a previous uterine scar. The results of this evaluation therefore carry important implications for future research but cannot represent true cost-effectiveness.

Economic evaluations are undertaken to inform decision making, whether it is to determine the effectiveness of a new drug or health technology or the cost-effectiveness of an existing treatment. Cost-effectiveness analysis (CEA) is a particular type of economic evaluation that compares the costs and effects of two or more comparators [16]. The outcome measurement is expressed in terms of an incremental cost-effectiveness ratio (ICER). Using standard cost-effectiveness analysis methods, the aim of this study was to examine the costs and short-term maternal outcomes associated with a TOLAC and an ERCD for a hypothetical cohort of low risk women. Low risk women were defined according to the National Institute for Health and Clinical Excellence (NICE) guidelines on intrapartum care [31]. The study assumed the perspective of the health system in Ireland and considered a six week time horizon. This allowed for variations in health-related quality of life arising from maternal complications and each delivery pathway, with average postpartum recovery time rarely exceeding three weeks, while also allowing for one follow-up consultation in the event of puerperal infection and its associated impact on health-related quality of life.

## Methods

**Figure 1:**
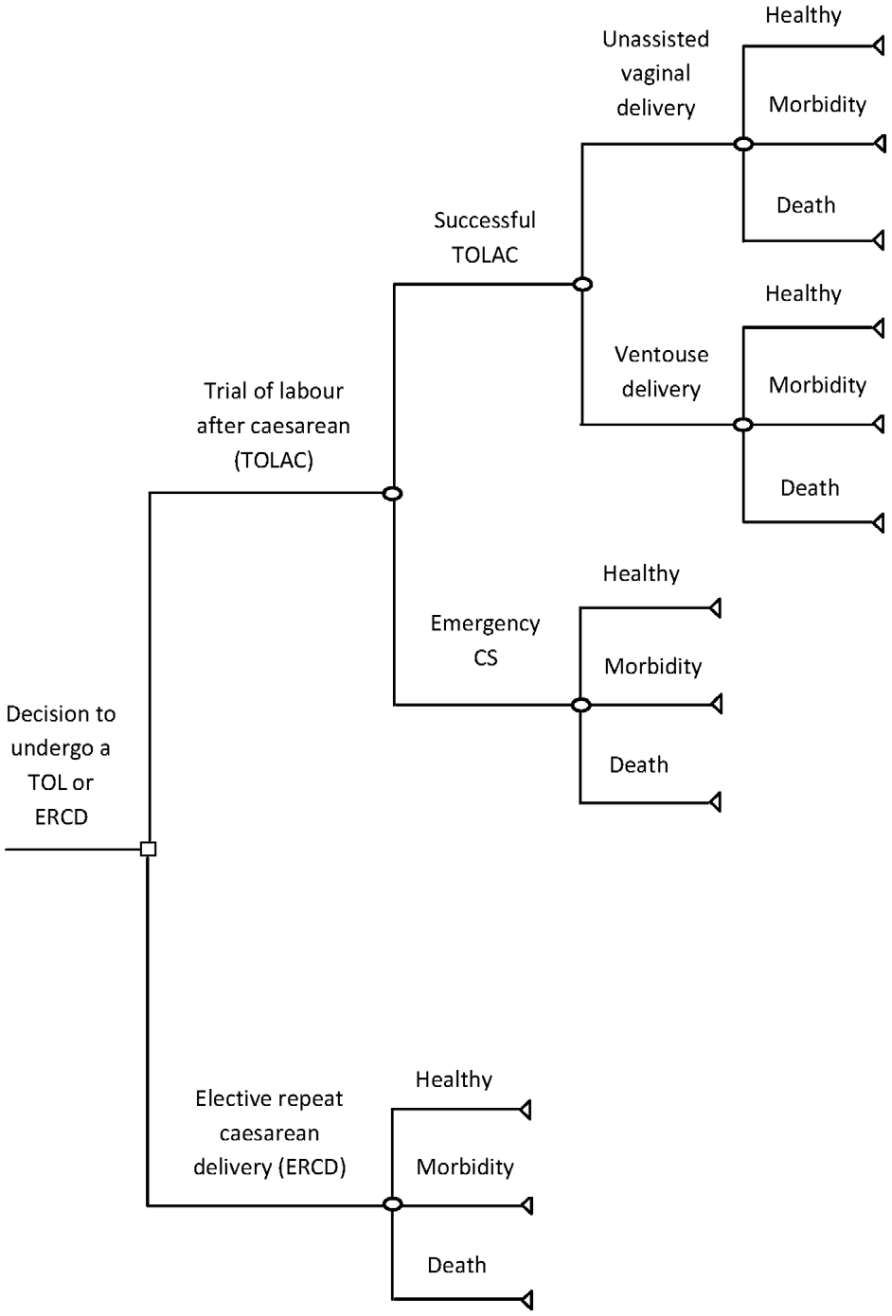
Decision tree representing all possible consequences arising from the decision to undergo a TOLAC or ERCD.

To compare the costs and consequences of a TOLAC compared with an ERCD, a decision analytic model was employed. Decision modelling can be defined as the systematic approach to decision-making under conditions of uncertainty [27]. In a decision analytic model, consequences are expressed as probabilities, weighted against costs and outcomes to derive an expected value for each alternative option. To represent the model, thus capturing all possible consequences that could flow from the decision to undergo a TOLAC or an ERCD, a decision-tree model was used (Figure 1). In this model, a woman could have a successful TOLAC or fail the trial, resulting in an emergency caesarean delivery. A successful TOLAC encompassed both unassisted and assisted vaginal deliveries – ventouse delivery was modelled as an assisted vaginal delivery because it is more commonly used in Ireland, accounting for 12.2% of all deliveries in 2009 [32]. In either case, a woman could have a complication-free delivery (‘*Healthy*’), suffer a maternal morbidity (‘*Morbidity*’), or die (‘*Death*’); the same maternal outcomes arose following an emergency caesarean section and ERCD. Using a Bayesian technique, maternal complications were grouped into one health state where the probability of an event was weighted according to its proportional size to overall morbidities [27].

In order to complete a decision analytic model, data on TOLAC success rates, potential adverse health events, and delivery costs were required. Information on TOLAC success rates was available from seven of the 20 maternity hospitals in Ireland, representing 61.6% of all deliveries in the country in 2009. The data are assumed representative of the general obstetric population as more than half of all births were captured by the seven hospitals during this period. Of the group attempting a TOLAC in 2009, two-thirds (66.6%) were successful. While the rate of ventouse deliveries in the TOLAC group was unavailable in individual hospital reports, nationally representative hospital discharge data found that 12.2% of all deliveries were ventouse deliveries during 2009 [32]. Since the rate may be higher in women attempting a TOLAC, in the decision tree, a base case estimate of 13% was assumed. Variation in the rate of assisted deliveries was accounted for in the probabilistic sensitivity analysis. A hypothetical cohort of 10,000 low risk women was used in the decision analytic model to closely resemble morbidity patterns in a small population.

Because there are no published Irish data on maternal morbidity following a TOLAC or an ERCD, incidence rates from a recent systematic review conducted in North America [2] were used to derive the probability of an adverse event. The model considered five major maternal complications which are commonly associated with a TOLAC and an ERCD and maternal mortality. These morbidities included uterine rupture, hysterectomy, operative injury, blood transfusion, and postpartum endometritis.

Cost data were compiled from primary data collection and combined both “bottom-up” and “top-down” costing techniques. As the study assumed the perspective of the health system, only direct costs to the government were included. All unit costs were expressed in Euro and valued at 2010 prices. Due to the short-term nature of the economic evaluation, discounting was exempt from the analysis as costs and outcomes accrued immediately rather than in the future.

To identify all resources used during a TOLAC and an ERCD along with other procedures, a resource use inventory was developed and approved by a health economist (CGF), a clinical manager midwife (CME) and a consultant obstetrician (RAG). The inventory identified all resources used during the various procedures and alternative delivery pathways, including medical supplies, pharmaceuticals, blood units, and time spent by each healthcare professional with the woman in each instance. The following procedures were micro-costed: epidural and spinal injections, general anaesthetic, vaginal birth and ventouse birth, caesarean section (elective and emergency), uterine rupture, hysterectomy, operative injury, blood transfusion, endometritis, and maternal mortality. The cost of an epidural was included in the cost of a TOLAC because it is administered in approximately 60% of deliveries in Ireland (unpublished data; derived from the Hospital In-Patient Enquiry (HIPE) scheme, a computerised database that records hospital activity). Augmentation costs were excluded, however, as induction is discouraged in women with a previous caesarean delivery [31]. The average duration of labour following a successful TOLAC, calculated from a case-control study (conducted in a teaching hospital; results to be published), was estimated at 7.5 hours in the base case. For the emergency caesarean section group, a five-hour duration of labour was assumed according to expert opinion. Variation in each of these parameters was accounted for in the probabilistic sensitivity analysis.

Consideration was also given to administrative costs (staff costs), operational costs (overheads such as heating and lighting, building maintenance), capital costs (land and building), and extended length of stay costs. Administrative costs for midwives and clinicians were obtained from consolidated salary scales [29]. Associated non-pay costs, including employer’s PRSI contributions, superannuation, and overheads, were also estimated [33], [34].

Length of stay differed according to mode of delivery and maternal morbidity status. Estimated length of stay during 2005–2009 was derived from hospital discharge data (data derived from HIPE). Median length of hospital stay was two days following a successful vaginal delivery; three days following a ventouse delivery and five days following a caesarean delivery. Length of stay succeeding each maternal complication varied from five days for uterine rupture and blood transfusion to six days following an operative injury to 11 days for a hysterectomy. Representing the top-down costing estimation, costs per bed-day were applied where duration of stay exceeded two days since two days were common to both groups. Bed-day costs were based on relevant Diagnostic Related Groups, which represents groups of patients who share similar clinical attributes and consume similar levels of resources. The following DRG codes were used: DRG (O60B; O01C; O02A; O01B).

As a measure of health gain, the cost-effectiveness analysis used health-related quality of life, calculated as quality-adjusted life years (QALY). A QALY can be described as a composite measure of both length of life and health-related quality of life that can be impacted by health care programmes and interventions [16]. It is a generic measure of health which is widely used in clinical areas to compare across various disease and illness conditions. A QALY is typically calculated using a linear scale with two discrete points, 0.00 (death) and 1.00 (perfect health), where health-related quality of life (also known as utility or weight) is assumed constant between each interval. Taken from Chung *et al* [22], Quality of Well-Being community preference weights were applied to represent the disutility associated with each delivery pathway and complication [30]. These weights described the scaled reduction in quality of life across four dimensions of health: symptom complexes, mobility, physical activity, and social activity. A modified weight and duration of disutility was given to each dimension, according to updated scales (Table 1). For example, the study assumed that an unassisted vaginal delivery was associated with a disutility of 0.41, lasting seven days, while ERCD was associated with a disutility of 0.58, lasting three weeks. QALYs were subsequently calculated by multiplying the utility of each condition by the associated duration of disutility, dependent on the study’s six week time frame. For instance, following a successful vaginal delivery, a woman’s health related quality of life yielded 0.93 QALYs over a six week time frame or 0.99 QALYs over one year. A cost-effectiveness threshold of €45,000 per QALY was applied in accordance with a historical and notional Irish cost-effectiveness threshold [35].

**Table 1:** Disutilities for each delivery pathway and complication.

Health state	QWB components				Disutilityper day	Duration(days)
	CPX	MOB	PAC	SAC		
Successful TOLAC	0.256	0.031	0.072	0.054	0.41	7
Emergency CS	0.424	0.031	0.072	0.054	0.58	21
ERCD	0.424	0.031	0.072	0.054	0.58	21
Uterine rupture	0.424	0.031	0.072	0.054	0.58	21
Hysterectomy	0.424	0.031	0.072	0.054	0.58	21
Operative injury	0.369	0.031	0.072	0.054	0.53	21
Blood transfusion	0.256	0.031	0.072	0.054	0.41	7
Endometritis	0.160	0.089	0.072	0.054	0.38	14

Source: QWB-SA scale (2008).

Abbreviations: CPX, symptom complexes; MOB, mobility; PAC, physical activity, SAC, social activity; TOLAC, trial of labour after caesarean; CS, Caesarean section; ERCD, Elective repeat Caesarean delivery.

An incremental cost-effectiveness ratio (ICER) was estimated using the expected costs and effects of a TOLAC and an ERCD. The estimated ICER represented the study’s base case cost-effectiveness results or the deterministic model’s cost-effectiveness results. Probabilistic sensitivity analysis was also performed to account for parameter uncertainty. To do so, probability distributions were assigned to the individual model parameters (see Table S1). Costs and utilities assumed normal probability distributions because the data informing the parameters were unknown, whereas decision tree transition probabilities assumed beta distributions because the input parameters were binomial. (The range of the parameter estimates were typically determined by the standard error of each input parameter; however, where the standard error could not be identified, estimated figures were assumed *a priori*, and a number of validation exercises were performed to assess model stability). A Monte Carlo simulation with 10,000 iterations was performed using Microsoft Excel software (Microsoft, Seattle, WA). This simulation propagated the uncertainty in the individual model parameters, reflected by the assigned probability distributions, through the model to produce a distribution of expected costs and effects associated with each delivery mode. The results of the simulation were plotted on an incremental cost-effectiveness plane where costs are plotted on the north-south axis and effects are plotted on the east-west axis. An incremental cost-effectiveness plane describes four quadrants. To the north-east and south-west quadrant, an ICER is generated where the costs and effects of the intervention are either higher (north-east) or lower (south-west) than the control – a trade-off is required. The north-west quadrant indicates higher costs but lower effects such that the intervention is said to be dominated by the control, while the south-east quadrant illustrates lower costs and greater effects, where the intervention is said to dominate the control [16]. The ICER was calculated using mean values of the distributions of expected costs and effects.

## Results

### Costs and Maternal Outcomes

The cost of an unassisted vaginal delivery in Ireland was estimated at €627.94 (Table 2). This estimation represents the micro-costing of medical consumables, pharmaceuticals, and medical staff. A ventouse delivery, which is associated with an extended duration of stay, was estimated at €1,637.09. A modest difference between the estimated cost of an emergency caesarean section (€4,423.39) and an elective caesarean (€4,095.01) was identified. However, both procedures differed considerably in terms of staff costs as emergency caesarean section was associated with increased staff costs due to extended duration of labour and the increased necessity for the presence of specialised medical staff at delivery, such as a neonatologist.

**Table 2:** Estimated cost data for each delivery pathway and complication.

	Successful TOLACunassisted	Successful TOLAC ventouse	Emergency CS	ERCD
Delivery costs				
Cost of medical consumables	€104.44*	€180.34*	€173.06	€130.68
Staff costs	€523.50	€573.75	€767.33	€481.33
Average length of stay (HIPE)	2 days	3 days	5 days	5 days
DRG cost per bed-day	n/a	€883	€1,161	€1,161
Total cost per woman (€)	€627.94	€1,637.09	€4,423.39	€4,095.01
Complication costs	Cost excluding mode of delivery			
Uterine rupture		€1,235.33		
Hysterectomy		€905.94		
Operative injury		€355.25		
Blood transfusion		€596.63		
Endometritis		€49.50		

Abbreviations: TOLAC, trial of labour after caesarean; CS, Caesarean section; ERCD, Elective repeat Caesarean delivery; HIPE, Hospital in-patient enquiry scheme.

* This includes cost of epidural.

The decision analytic model evaluated maternal outcomes among a hypothetical cohort of 10,000 women in each arm. Following the decision to undergo a TOLAC, two-thirds of women had a vaginal delivery (6,664) (Table 3). Of this group, 6,521 women (98%) had a complication-free vaginal delivery, while 143 women (2%) experienced a maternal morbidity. Some 867 women (13%) had a ventouse delivery. Uterine rupture occurred in 24 instances across unassisted and assisted vaginal deliveries, while endometritis arose in 87 cases. Following a failed TOLAC, 465 women (14%) experienced an obstetric complication; uterine rupture occurred in 69 deliveries; operative injury occurred in 91 deliveries; endometritis occurred in 277 deliveries. In the ERCD group, uterine rupture was non-existent, while the frequency of other morbidities rate was less than the TOLAC group. Overall, maternal morbidities in the ERCD group (290 women) accounted for less than half the number of morbidities in the TOLAC group (608 women). However, in one instance a maternal death occurred in the ERCD group, but was essentially absent following a TOLAC.

**Table 3:** Distribution of maternal outcomes following a TOLAC and ERCD.

	Successful TOLAC(n = 6,664)			
Maternal outcome	Unassisted (n = 5,797)	Ventouse (n = 867)	Emergency CS(n = 3,336)	ERCD(N = 10,000)
Healthy	5,673 (98)	848 (98)	2,871 (86)	9,709 (97)
Total morbidity	124 (2)	19 (2)	465 (14)	290 (3)
Uterine rupture	21 (17)	3 (16)	69 (15)	0 (0)
Hysterectomy	5 (4)	1 (5)	7 (1)	11 (4)
Operative injury	4 (3)	1 (5)	91 (20)	56 (19)
Blood transfusion	19 (15)	3 (16)	20 (4)	27 (9)
Endometritis	76 (61)	11 (58)	277 (60)	196 (68)
Maternal mortality	0 (0)	0 (0)	0 (0)	1 (0)

### Deterministic Model

Despite a modestly higher rate of maternal morbidity following a TOLAC, the results of the decision analytic model suggest that a TOLAC is the cost-effective method of delivery for low risk women. The main advantages of a TOLAC were realised in the reduced length of stay in hospital and higher utility following a vaginal delivery. In terms of health improvements, a TOLAC generated 0.84 QALYs for a woman over the six week time frame, while an ERCD was associated with 0.70 QALYs, accounting for an incremental benefit of 0.14 QALYs (Table 4). This is based on the total amount of QALYs available within a six week time frame, or 0.1 of 0.12 QALYs available in a given year for a woman in the TOLAC group. While the incremental effect is slight, the difference in cost is considerable. The expected cost of a TOLAC was €1,835.06 per woman, opposed to €4,039.87 for an ERCD.

**Table 4:** Cost-effectiveness results of a TOLAC versus an ERCD.

	TOLAC		ERCD		ICER
Model	Cost (€)	QALYs	Cost (€)	QALYs	(€/QALY)
Deterministic	€1,830.73	0.84	€4,039.87	0.70	TOL dominates
Probabilistic	€1,833.28	0.84	€4,041.54	0.70	TOL dominates

Abbreviations: TOLAC, trial of labour after caesarean; ERCD, elective repeat Caesarean delivery; ICER, incremental cost-effectiveness ratio; QALY, quality-adjusted life year.

### Probabilistic Sensitivity Analysis

**Figure 2:**
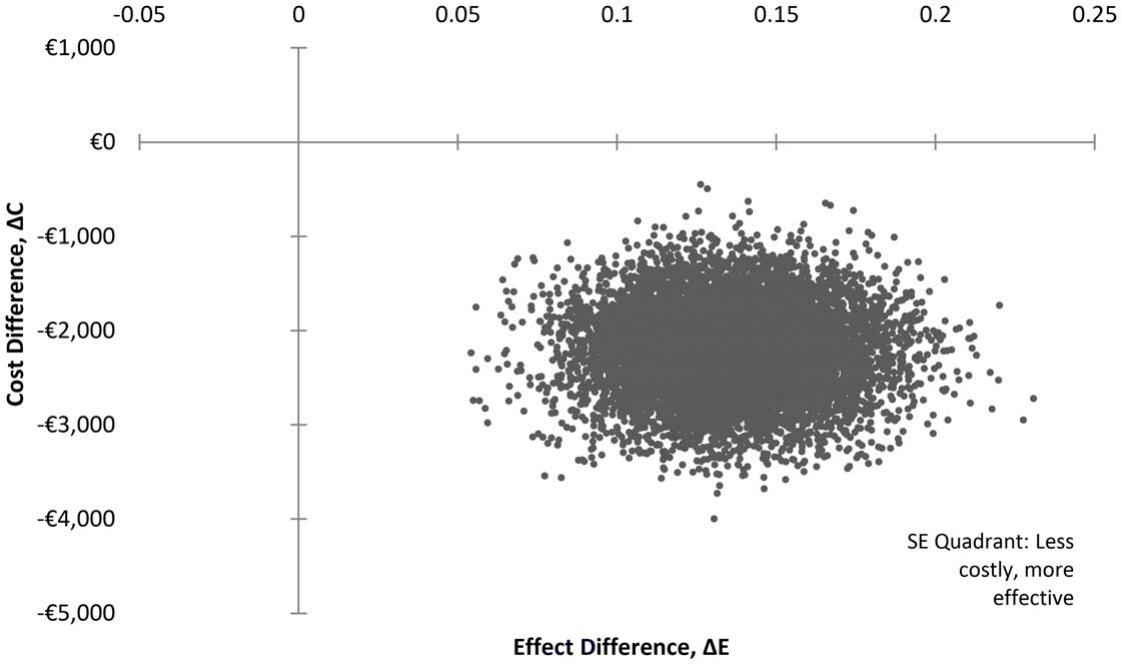
Results of 10,000 Monte Carlo simulations on the cost effectiveness plane for a TOLAC and ERCD.

The aim of probabilistic sensitivity analysis was to examine the existence and extent of uncertainty in the input parameters and, hence, expected values. Results from the Monte Carlo simulations (the point- and interval-estimate values used in the probabilistic sensitivity analysis are detailed in Table S2), which are plotted in the south-east quadrant of the incremental cost-effectiveness plane (Figure 2), confirmed that a TOLAC was both less costly and more effective than an ERCD. The expected cost of a TOLAC was estimated at €1,833.56, yielding 0.84 QALYs per woman, while an ERCD was estimated at €4,038.40 with an expected effect of 0.70 QALYs. Accordingly, the control group was said to be dominated by the study’s intervention. This is illustrated in the cost-effectiveness plane where individual trials do not cross the vertical or horizontal axes. As such, the probability that a TOLAC was cost-effective was 100 per cent.

## Discussion

To our knowledge, this is the first detailed analysis comparing the cost-effectiveness of a TOLAC to an ERCD in Europe (based on the Euro currency). This study enhances existing costing estimations as it is the first to rigorously employ a “bottom-up” costing technique in its evaluation of delivery and complication costs. Estimated costs derive from an exhaustive costing process, and may subsequently become the benchmark for costing obstetric procedures and alternative deliveries in Ireland and Europe. Further, we are unaware of any other study to date that has incorporated the cost of an assisted delivery in its comparison with ERCD.

Our hypothetical model found that a TOLAC was both less costly and more effective than an ERCD, despite an increased risk of maternal morbidity following a TOLAC. The main advantages of a successful TOLAC were realised in the reduced length of stay in hospital and higher utility following a vaginal delivery relative to an ERCD.

Despite using a hypothetical model, our costing for a vaginal delivery (€627.94) is in line with previous Irish reports (€631.64) [36]. Moreover, our findings that a TOLAC is more cost-effective than an ERCD were in accordance with research from the US [21], [22]. However, there were notable differences between the studies. Chung *et al* [22] found that a TOLAC was the most cost-effective method of delivery if the probability of successful TOLAC was at least 74%. In contrast, this study found that a TOLAC was cost-effective if the probability of success was 67%. Varying the success rate between 64% and 69% did not alter the cost-effectiveness results (see Tables S1–S2 for further information on parameter ranges). Nonethless, these differences in the required success threshold may be attributed in part to disparate costing estimations. For instance, our study assumed the perspective of the health system, while Chung *et al* [22] assumed a much broader perspective, that of society. Grobman *et al* [21] also found that a TOLAC was the cost-effective method of delivery relative to an ERCD. Similar to our study, the results of the cost-effectiveness analysis were robust to changes in key variables, such as cost and probability variables.

Our study has several limitations. Firstly, our findings were based on a hypothetical model and only focused on the major short-term maternal complications arising from a TOLAC and an ERCD. Other obstetric complications, such as thromboembolic disease [37] and urinary incontinence [38], were not included due to limited availability of published data. Moreover, inclusion of potential adverse neonatal outcomes and long-term maternal outcomes, such as increased risk of perinatal death, cerebral palsy, sub-fertility [39], [40] or placenta accreta [41], would have been beyond the capabilities of the model and study timeline. These issues should be explored in future research, where subsequent iterations synthesize relevant evidence under a new and comprehensive decision model. Evidence synthesis plays a key role in reducing uncertainty in decision making and should be the focus of future economic evaluations. Until the level of uncertainty in the expected cost and clinical outcome is minimised, true cost-effectiveness cannot be represented.

Secondly, maternal morbidity rates following a TOLAC versus ERCD for our model were based on research from North America, and thus may not be fully generalisable to an Irish setting. For example, the prevalence of obesity in the US is considerably higher than in Ireland [42], which may directly impact the frequency of uterine rupture [43] and subsequently overestimate its incidence in our study. Still, overestimating uterine rupture incidence would only strengthen our conclusion that a TOLAC is more cost-effective than an ERCD. In addition, while we extracted our rates from a systematic review which predominantly focused on morbidity rates in the USA, reassuringly, these rates are similar to those reported in other developed countries, such as the UK and Australia [44]–[48].

Also, the model does not allow for comorbidities. While probabilistic sensitivity analysis appropriately accounts for variations in each of the input parameters, it cannot account for potential comorbidities within the cohort model. At present, the incidence of comorbidities is not well documented, and research is needed to determine the likelihood of multiple morbidities in women with a previous caesarean section.

Lastly, with no utility index currently designed to measure health gain following childbirth and obstetric complications, off-the-shelf weights were applied to derive disutilities for vaginal and caesarean deliveries. There is an inherent weakness in applying community derived weights to represent health gain following childbirth. A more rigorous utility index is ideal and should be sought for future research.

Nevertheless, our results are timely and carry important economic and public health implications. Given the substantial increase in caesarean delivery across Europe over the past decade, clinicians need to be well informed of both the short-term and long-term benefits and risks of a TOLAC. Vaginal delivery should be suggested as a birth option for low risk women where success of a TOLAC is likely. Ideally, clinician-patient discourse would address differences in length of hospital stay and postpartum recovery time. While it is premature to advocate an institutional policy of TOLAC, local maternity units and clinical managers should be encouraged to evaluate their own rates of ERCD and attempted TOLAC. Furthermore, to better understand barriers in attempted TOLAC, mixed methods research focused on clinician and patient preferences regarding mode of delivery should be undertaken.

## Supporting Information

Table S1 Parameter Statistics and Distributions for Probabilistic Sensitivity Analysis.
(DOC)

Table S2 Probability and interval estimates used in probabilistic sensitivity analysis.
(DOC)
