# Supplementary material for: At What Price? A Cost-Effectiveness Analysis Comparing Trial of Labour after Previous Caesarean versus Elective Repeat Caesarean Delivery
Source: PLoS One. 2013 Mar 6;8(3):e58577. doi: 10.1371/journal.pone.0058577 (PMC3590223; doi:10.1371/journal.pone.0058577)
Supplement: Table S1 — Parameter Statistics and Distributions for Probabilistic Sensitivity Analysis. (DOC) [file pone.0058577.s001.doc]

| **Table S1: Parameter Statistics and Distributions for Probabilistic Sensitivity Analysis** | | | | | |
| --- | --- | --- | --- | --- | --- |
| **Description** | **Mean** | **Std error** | **alpha** | **beta** | **distribution** |
| **Decision tree transition probabilities** | |  |  |  |  |
| Probability of successful TOLAC | 0.6664 |  | 1516 | 759 | Beta |
| Probability of emergency CS | 0.3336 |  |  |  | Beta |
| Probability of unassisted delivery | 0.87 |  |  |  | Normal |
| Probability of ventouse delivery | 0.13 | 0.04 |  |  | Normal |
| VBAC |  |  |  |  |  |
| Morbidity | 0.0214 |  |  |  |  |
| Death | 0 |  |  |  | Beta |
| Healthy | 0.9786 |  |  |  | Beta |
| Uterine rupture | 0.0036 |  | 62 | 17273 | Beta |
| Hysterectomy | 0.0008 |  | 90 | 109210 | Beta |
| Operative injury | 0.0006 |  | 2 | 3286 | Beta |
| Blood transfusion | 0.0033 |  | 365 | 110261 | Beta |
| Endometritis | 0.0131 |  | 192 | 14432 | Beta |
| **Emergency caesarean section** |  |  |  |  |  |
| Morbidity | 0.1394 |  |  |  | Beta |
| Death | 0.0001 |  |  |  | Beta |
| Healthy | 0.8605 |  |  |  | Beta |
| Uterine rupture | 0.0208 |  | 142 | 6675 | Beta |
| Hysterectomy | 0.0021 |  | 94 | 43887 | Beta |
| Operative injury | 0.0274 |  | 45 | 1597 | Beta |
| Blood transfusion | 0.0061 |  | 269 | 44067 | Beta |
| Table S1 continued |  |  |  |  |  |
| **Description** | **Mean** | **Std error** | **alpha** | **beta** | **distribution** |
| Endometritis | 0.083 |  | 444 | 4908 | Beta |
| **Elective repeat caesarean delivery** |  |  |  |  |  |
| Morbidity | 0.029 |  |  |  | Beta |
| Death | 0.0001 |  |  |  | Beta |
| Healthy | 0.9709 |  |  |  | Beta |
| Uterine rupture | 0 |  | 1 | 20328 | Beta |
| Hysterectomy | 0.0011 |  | 217 | 203404 | Beta |
| Operative injury | 0.0056 |  | 23 | 4074 | Beta |
| Blood transfusion | 0.0027 |  | 550 | 204279 | Beta |
| Endometritis | 0.0196 |  | 332 | 16627 | Beta |
|  |  |  |  |  |  |
|  |  |  |  |  |  |
| **Event disutilities** |  |  |  |  |  |
| VBAC | 0.413 | 0.04 |  |  | Normal |
| Em. CS | 0.581 | 0.05 |  |  | Normal |
| ERCD | 0.581 | 0.04 |  |  | Normal |
| Uterine rupture | 0.581 | 0.05 |  |  | Normal |
| Hysterectomy | 0.581 | 0.05 |  |  | Normal |
| Operative injury | 0.526 | 0.04 |  |  | Normal |
| Blood transfusion | 0.413 | 0.03 |  |  | Normal |
| Endometritis | 0.375 | 0.03 |  |  | Normal |
| Table S1 continued |  |  |  |  |  |
| **Description** | **Mean** | **Std error** | **alpha** | **beta** | **distribution** |
| **Event costs** |  |  |  |  |  |
| **TOLAC** |  |  |  |  |  |
| **Unassisted delivery** | €627.94 | €180.00 |  |  | Normal |
| Uterine rupture | €5,199.27 | €490.00 |  |  | Normal |
| Hysterectomy | €11,483.38 | €730.00 |  |  | Normal |
| Operative injury | €5,418.19 | €540.00 |  |  | Normal |
| Blood transfusion | €4,531.32 | €470.00 |  |  | Normal |
| Endometritis | €677.44 | €180.00 |  |  | Normal |
| Maternal mortality | €139.75 | €40.00 |  |  | Normal |
| **Ventouse delivery** | €1,637.09 | €330.00 |  |  |  |
| Uterine rupture | €5,325.42 | €530.00 |  |  | Normal |
| Hysterectomy | €11,609.53 | €750.00 |  |  | Normal |
| Operative injury | €5,544.34 | €550.00 |  |  | Normal |
| Blood transfusion | €4,657.47 | €480.00 |  |  | Normal |
| Endometritis | €1,686.59 | €350.00 |  |  | Normal |
| Maternal mortality | €265.90 | €60.00 |  |  | Normal |
| **Emergency caesarean section** |  |  |  |  |  |
| Em. CS | €4,306.39 | €450.00 |  |  | Normal |
| Uterine rupture | €6,114.72 | €580.00 |  |  | Normal |
| Hysterectomy | €14,378.83 | €780.00 |  |  | Normal |
| Operative injury | €6,813.64 | €630.00 |  |  | Normal |
| Table S1 continued |  |  |  |  |  |
| **Description** | **Mean** | **Std error** | **alpha** | **beta** | **distribution** |
| Blood transfusion | €5,626.77 | €530.00 |  |  | Normal |
| Endometritis | €4,355.89 | €440.00 |  |  | Normal |
| Maternal mortality | €4,095.01 | €50.00 |  |  | Normal |
| **Elective repeat caesarean delivery** |  |  |  |  |  |
| ERCD | €4,095.01 | €430.00 |  |  | Normal |
| Uterine rupture | €5,903.34 | €550.00 |  |  | Normal |
| Hysterectomy | €14,167.45 | €770.00 |  |  | Normal |
| Operative injury | €6,602.26 | €610.00 |  |  | Normal |
| Blood transfusion | €5,415.39 | €510.00 |  |  | Normal |
| Endometritis | €4,144.51 | €450.00 |  |  | Normal |
| Maternal mortality | €4,306.39 | €70.00 |  |  | Normal |
