# Supplementary material for: At What Price? A Cost-Effectiveness Analysis Comparing Trial of Labour after Previous Caesarean versus Elective Repeat Caesarean Delivery
Source: PLoS One. 2013 Mar 6;8(3):e58577. doi: 10.1371/journal.pone.0058577 (PMC3590223; doi:10.1371/journal.pone.0058577)
Supplement: Table S2 — Probability and interval estimates used in probabilistic sensitivity analysis. (DOC) [file pone.0058577.s002.doc]

| **Table S2:** Probability and interval estimates used in probabilistic sensitivity analysis | | | | | |
| --- | --- | --- | --- | --- | --- |
|  |  | | **Range** | | |
| **Description** | **Point-estimate** | | **2.5% Percentile** | | **97.5% Percentile** |
| **Decision tree transition probabilities** | | |  | |  |
| Probability of successful TOLAC | | 0.67 | 0.65 | | 0.69 |
| Probability of emergency CS | | 0.33 | 0.31 | | 0.35 |
| Probability of unassisted delivery | | 0.87 | 0.79 | | 0.95 |
| Probability of ventouse delivery | | 0.13 | 0.05 | | 0.21 |
| **Successful TOLAC** | |  |  | |  |
| Morbidity | | 0.02 | 0.02 | | 0.02 |
| Death | | 0.00 | 0.00 | | 0.00 |
| Healthy | | 0.98 | 0.98 | | 0.98 |
| Uterine rupture | | 0.17 | 0.13 | | 0.21 |
| Hysterectomy | | 0.04 | 0.03 | | 0.05 |
| Operative injury | | 0.03 | 0.00 | | 0.08 |
| Blood transfusion | | 0.15 | 0.13 | | 0.18 |
| Endometritis | | 0.61 | 0.56 | | 0.66 |
| **Emergency caesarean section** | |  |  | |  |
| Morbidity | | 0.14 | 0.13 | | 0.15 |
| Death | | 0.00 | 0.00 | | 0.00 |
| Healthy | | 0.86 | 0.85 | | 0.87 |
| Uterine rupture | | 0.15 | 0.13 | | 0.17 |
| Hysterectomy | | 0.02 | 0.01 | | 0.02 |
| Operative injury | | 0.20 | 0.15 | | 0.24 |
| Blood transfusion | | 0.04 | 0.04 | | 0.05 |
| Table S2 continued |  | | **Range** | | |
| **Description** | **Point-estimate** | | **2.5% Percentile** | **97.5% Percentile** | |
| Endometritis | | 0.60 | 0.55 | | 0.64 |
| **Elective repeat caesarean delivery** | | |  | |  |
| Morbidity | 0.03 | | 0.03 | | 0.03 |
| Death | 0.00 | | 0.00 | | 0.00 |
| Healthy | 0.97 | | 0.97 | | 0.97 |
| Uterine rupture | 0.00 | | 0.00 | | 0.01 |
| Hysterectomy | 0.04 | | 0.03 | | 0.04 |
| Operative injury | 0.19 | | 0.13 | | 0.26 |
| Blood transfusion | 0.09 | | 0.08 | | 0.11 |
| Endometritis | 0.68 | | 0.62 | | 0.73 |
| **Event disutilities** |  | |  | |  |
| TOLAC | 0.41 | | 0.33 | | 0.49 |
| Uterine rupture | 0.10 | | 0.08 | | 0.11 |
| Hysterectomy | 0.02 | | 0.02 | | 0.03 |
| Operative injury | 0.01 | | 0.01 | | 0.02 |
| Blood transfusion | 0.06 | | 0.05 | | 0.07 |
| Endometritis | 0.23 | | 0.23 | | 0.23 |
| Em. CS | 0.58 | | 0.48 | | 0.68 |
| Uterine rupture | 0.09 | | 0.07 | | 0.10 |
| Hysterectomy | 0.01 | | 0.01 | 0.01 | |
| Operative injury | 0.10 | | 0.09 | 0.12 | |
| Blood transfusion | 0.02 | | 0.02 | 0.02 | |
| Endometritis | 0.22 | | 0.19 | 0.26 | |
| Table S2 continued |  | | **Range** | | |
| **Description** | **Point-estimate** | | **2.5% Percentile** | **97.5% Percentile** | |
| ERCD | 0.58 | | 0.50 | 0.66 | |
| Uterine rupture | 0.00 | | 0.00 | 0.00 | |
| Hysterectomy | 0.02 | | 0.02 | 0.02 | |
| Operative injury | 0.10 | | 0.09 | 0.12 | |
| Blood transfusion | 0.04 | | 0.03 | 0.04 | |
| Endometritis | 0.25 | | 0.21 | 0.29 | |
| Death | 0.00 | | 0.00 | 0.00 | |
| Event costs |  | |  |  | |
| **TOLAC** |  | |  |  | |
| Unassisted delivery | €628.44 | | €280.14 | €983.11 | |
| Uterine rupture | €872.38 | | €714.62 | €1,035.58 | |
| Hysterectomy | €440.98 | | €387.44 | €495.15 | |
| Operative injury | €153.33 | | €123.37 | €182.98 | |
| Blood transfusion | €696.82 | | €555.73 | €833.84 | |
| Endometritis | €413.81 | | €196.81 | €628.14 | |
| Maternal mortality | €140.13 | | €61.42 | €218.29 | |
| Ventouse delivery | €1,637.66 | | €974.45 | €2,278.52 | |
| Uterine rupture | €894.49 | | €722.64 | €1,067.87 | |
| Hysterectomy | €444.82 | | €387.79 | €501.78 | |
| Operative injury | €156.98 | | €127.21 | €187.32 | |
| Blood transfusion | €715.99 | | €572.49 | €856.83 | |
| Endometritis | €1,029.86 | | €607.81 | €1,446.94 | |
| Maternal mortality | €265.70 | | €148.22 | €384.32 | |
| Table S2 continued |  | | **Range** | | |
| **Description** | **Point-estimate** | | **2.5% Percentile** | **97.5% Percentile** | |
| **Emergency caesarean section** | | |  |  | |
| Em. CS | €4,421.19 | | €3,546.73 | €5,303.70 | |
| Uterine rupture | €912.19 | | €739.56 | €1,082.07 | |
| Hysterectomy | €220.37 | | €196.73 | €243.63 | |
| Operative injury | €1,338.89 | | €1,098.97 | €1,581.12 | |
| Blood transfusion | €244.95 | | €200.11 | €289.68 | |
| Endometritis | €2,593.64 | | €2,077.76 | €3,106.55 | |
| Maternal mortality | €572.85 | | €475.78 | €670.50 | |
| **Elective repeat caesarean delivery** | | |  |  | |
| ERCD | €4,096.38 | | €3,260.88 | €4,937.46 | |
| Uterine rupture | €10.02 | | €8.21 | €11.83 | |
| Hysterectomy | €521.07 | | €464.85 | €578.83 | |
| Operative injury | €1,278.65 | | €1,047.57 | €1,509.87 | |
| Blood transfusion | €502.37 | | €410.66 | €596.24 | |
| Endometritis | €2,797.12 | | €2,202.76 | €3,388.32 | |
| Maternal mortality | €704.95 | | €568.41 | €843.91 | |
